# Core–Shell Nanostructured Drug Delivery Platform Based on Biocompatible Metal–Organic Framework-Ligated Polyethyleneimine for Targeted Hepatocellular Carcinoma Therapy

**DOI:** 10.1021/acsomega.3c01385

**Published:** 2023-05-31

**Authors:** Mostafa Fytory, Amira Mansour, Waleed M. A. El Rouby, Ahmed A. Farghali, Xiaorong Zhang, Frank Bier, Mahmoud Abdel-Hafiez, Ibrahim M. El-Sherbiny

**Affiliations:** † Nanomedicine Labs, Center for Materials Science (CMS), Zewail City of Science and Technology, 6 October City, 12578 Giza, Egypt; ‡ Material Science and Nanotechnology Department, Faculty of Postgraduate Studies for Advanced Sciences (PSAS), Beni-Suef University, 62511 Beni-Suef, Egypt; § Department of Physics and Astronomy, Uppsala University, Box 516, SE-75120 Uppsala, Sweden; ∥ Molecular Bioanalytics and Bioelectronics Group, Institute of Biochemistry and Biology, University of Potsdam, 14476 Potsdam-Golm, Germany

## Abstract

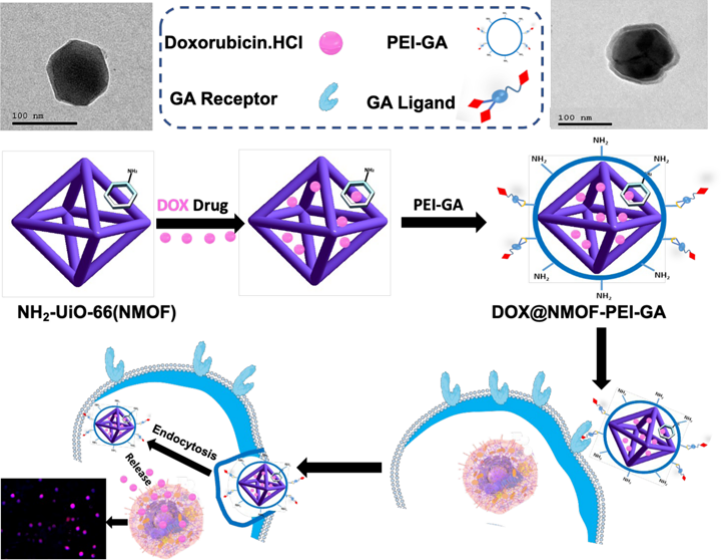

Multifunctional nanosized metal–organic frameworks (NMOFs) have advanced rapidly over the past decade to develop drug delivery systems (DDSs). These material systems still lack precise and selective cellular targeting, as well as the fast release of the quantity of drugs that are simply adsorbed within and on the external surface of nanocarriers, which hinders their application in the drug delivery. Herein, we designed a biocompatible Zr-based NMOF with an engineered core and the hepatic tumor-targeting ligand, glycyrrhetinic acid grafted to polyethyleneimine (PEI) as the shell. The improved core–shell serves as a superior nanoplatform for efficient controlled and active delivery of the anticancer drug doxorubicin (DOX) against hepatic cancer cells (HepG2 cells). In addition to their high loading capacity of 23%, the developed nanostructure DOX@NMOF-PEI-GA showed an acidic pH-stimulated response and extended the drug release time to 9 days as well as enhanced the selectivity toward the tumor cells. Interestingly, the DOX-free nanostructures showed a minimal toxic effect on both normal human skin fibroblast (HSF) and hepatic cancer cell line (HepG2), but the DOX-loaded nanostructures exhibited a superior killing effect toward the hepatic tumor, thus opening the way for the active drug delivery and achieving efficient cancer therapy applications.

## 1 Introduction

Nanosized metal–organic frameworks (NMOFs) have emerged as porous nanostructures with multiple applications.^1^ NMOFs have several distinctive advantages as malleable morphology, size, composition, and chemical characteristics while maintaining the desired physicochemical properties such as large surface area, high porosity, and uniformity.^2,3^ In addition to their tunable pore size and connectivities, they can display simultaneously hydrophobic and hydrophilic entities that fit the physicochemical characteristics of various drugs and their biomedical applications.^4^ Moreover, NMOFs can be used as drug delivery nanoplatforms for cancer treatment due to their high loading capacity, easy modification, and biocompatibility as well as their ability to be loaded with both hydrophilic and hydrophobic drugs.^5,6^ There are recent reports about the application of ligated NMOFs modified by one or more targeting ligands as platforms to develop active cancer drug delivery systems (DDSs).^7−9^ So, drug encapsulation into ligated NMOFs paved the way for enhanced targetability and improved bioavailability.^10^ Among all, zirconium (Zr)-based NMOFs of UiO-66 stand out from other nanomaterials owing to their outstanding chemical and thermal stabilities.^11^ Besides, Zr is biocompatible since it is daily consumed by humans (3.5 mg).^12^ The toxicity of nanosized NH_2_-UiO-66 and UiO-66 was investigated through an in vivo study. Remarkably, UiO-66, showed little cytotoxicity and biocompatibility as^13^ has been reported elsewhere.^14,15^ However, the active cancer targeting followed by controlling cargo release from NMOF carriers, as well as the fast degradation in the body’s metabolic system, still represents a challenge.

Therefore, coblending the high surface area, definite functionalities, and chemical control of the functional organic ligands with the corresponding characteristics of the other materials would improve the performance of nanomaterials in the field of biomedical applications.^16^ Besides, the merging of MOFs and specific polymers can develop a specific nanocomposite that leads to exploiting the profitable properties of both materials. These nanocomposites were introduced as core–shell nanostructures where MOFs are as the core while the polymers are acting as shells.^17^ The surface modification of NMOF with a natural polymer as a shell might introduce various advantages such as enhanced targetability, stability, and delayed NMOF biodegradation and consequently controlling the cargo release.^18^

Alginate is a biocompatible natural polysaccharide and is widely used in the pharmaceutical industry,^19^ mainly to achieve prolonged drug release. It contains carboxyl groups, and in acidic pH values, these groups are protonated and thus slow down the drug release.^20−22^ Besides, alginate has been used to coat drug-loaded nanoparticles as NMOF to achieve extended drug release in acidic pH, as reported by Vahed et al.^23^

Polyethyleneimine (PEI) has been widely used as a nonviral vector^24,25^ for cancer.^26,27^ PEI is a synthetic branched organic polycation polymer with a high density of amine groups and is characterized by its hydrophilicity and biocompatibility.^28^ As the pH decreases to 5, its protonation magnitude is enhanced to be 45%.^29^ In the acidic environment, as cancer and endosomes, PEI becomes positively charged by the proton sponge effect, which facilitates the cells’ endocytosis.^30^ Furthermore, PEI-based nanoparticles are considered of special interest in cancer drug delivery due to their penetration capability deep inside the solid tumors, which is attributed to the charge attraction between the positively charged polymers and the negatively charged channels of the vessels’ walls.^26^ Although the cytotoxicity of the branched PEI remains a great challenge, it is mitigated when hydrophobic units are grafted to the PEI backbone or when it is applied as coating due to the partial consumption of the positive charge. Additionally, the transfection efficiency is improved by altering the adsorption on the cell’s surface.^31,32^ Intriguingly, cationic polymers have been reported to be grafted with several molecules in order to achieve active drug delivery as glycyrrhetinic acid (GA).^33^ GA is a triterpenoid compound extracted from licorice.^34^ Due to the existence of GA receptors on the hepatocyte’s surface, it is an active hepatocyte targeting agent.^35,36^ Consequently, GA was reported to decorate several nanoparticle surfaces to achieve active drug delivery of anticancer agents to hepatocellular carcinoma (HCC).^37,38^ Remarkably, GA was used to graft PEI to selectively deliver the cargo to HepG2 cells with neglectable toxicity.^39^

One of the prevalent cancer types affecting human is hepatocellular carcinoma (HCC). Although the diagnosis and treatment approaches of HCC have been revolutionized, the off-target chemotherapy distribution still hampers clinically successful therapy.^40,41^ The anthracycline anticancer drug, doxorubicin (DOX), which is isolated from *Streptomyces peucetius*, has been widely used in the treatment of HCC. However, the cardiotoxicity was the main obstacle to administering DOX systemically.^42−44^ Consequently, several nanoparticles were reported for the selective delivery of DOX, particularly through decorating their surface with HCC-specific targeting ligands.^45^ To date, cancer therapy is mainly approached through passive targeting via enhanced permeability and retention (EPR) or active targeting of cancer cells through the anticancer drug loading into ligated nanocarriers, which paved the way toward enhanced selectivity and clinical outcome.^46^

In this work, we report the fabrication, characterization, and biological evaluation of newly developed core–shell nanostructures of DOX-loaded NMOF (DOX@NMOF) core coated with a ligated PEI shell. The core–shell nanostructures were developed for enhanced HCC-active targeting and efficient delivery of the drug DOX. The PEI was chemically conjugated with the GA targeting ligand via the formation of an amide bond between GA carboxylic groups and the PEI amine groups. On the other hand, the ligated PEI was linked electrostatically with the prepared DOX-loaded NMOF in the presence of alginate as a cross-linker. The newly developed DOX@NMOF-PEI-GA core–shell nanosystem was characterized, and its efficiency to selectively target the HCC cells was investigated by the cytotoxicity and cellular uptake assays.

## 2 Materials and Methods

### 2.1 Materials

2-Aminoterephthalic acid (99%) was purchased from Alfa Aesar (Germany). Zirconium tetrachloride (ZrCl_4_, 98%) and 18β-glycyrrhetinic acid (GA) were obtained from Acros-Organics (New Jersey). *N*-(3-Dimethylaminopropyl)-*N*-ethyl carbodiimide hydrochloride (EDC·HCl), *N*-hydroxy succinimide (NHS), branched PEI (25,000 kDa), doxorubicin hydrochloride (DOX·HCl), acetonitrile, and anhydrous *N*,*N*-dimethylformamide (DMF) were purchased from Sigma-Aldrich (Darmstadt, Germany). RPMI-1640, HEPES buffer solution, l-glutamine, gentamycin, and 0.25% trypsin–EDTA were obtained from Lonza (Belgium). All other used solvents were obtained from Alfa Aesar, A Johnson Matthey Company, U.K.

### 2.2 Methodology

#### 2.2.1 Synthesis of PEI-GA Conjugate

The PEI-GA conjugate was synthesized as reported elsewhere,^47^ with a minor modification, with a molar ratio of 1:0.25. Briefly, the GA moiety (270 mg, 0.25 mmol) in 10 mL of DMSO was activated by using EDC·HCl chemistry in the presence of NHS and the solution was vigorously stirred for 2 h at room temperature. Afterward, a solution of branched PEI 25 kDa (100 mg, 2.32 mmol) in 10 mL of DMSO was added and the mixture was left to be stirred for 72 h. The obtained solution was transferred into a dialysis bag (MWCO = 3500) and dialyzed against deionized water for 48 h. The resulting product was collected by centrifuge and freeze-dried.

#### 2.2.2 Synthesis of Nanosized NH_2_-UiO-66 NMOF and Loading with DOX

Nanosized NH_2_-UiO-66 (NMOF) was prepared as the earlier reported method.^48^ After the synthesis process, 100 mg of DOX was loaded into the NMOFs (details in SI.1 in the Supporting Information).

#### 2.2.3 Fabrication of Core–Shell Nanostructure of PEI-GA/Modified NH_2_-UiO-66 NMOF (NMOF-PEI-GA)

NMOF-PEI-GA core–shell nanostructure was obtained via the electrostatic interaction. Simply, 0.1 g of the prepared NMOF was dispersed with 50 mg of sodium alginate in aqueous acidic solution through vigorous stirring for 30 min. Afterward, a methanol solution of conjugated PEI-GA was added dropwise, and the mixture was left to stir overnight. The product was collected by centrifugation, washed three times with water, and then dried under vacuum.

#### 2.2.4 Physicochemical Characterization of the Developed NMOFs

Fourier transform infrared (FTIR) spectroscopy (VERTEX 70 FTIR spectrometer, Bruker Optics, Germany) was used to investigate the functional groups of the prepared NMOFs pre- and postformation of the core–shell to confirm the key transformations of the material. The instrument worked with a resolution of 4 cm^–1^ and 32 scans with a frequency range of 650–4000 cm^–1^. The surface ζ-potential of the various formulations was determined at 25 °C using electrophoretic light scattering (ELS) methods with the aid of a Malvern zetasizer (Malvern Instruments, Malvern, U.K.). The characteristics of the PEI-GA conjugate were examined by ^1^H nuclear magnetic resonance (^1^H NMR) (Avance 600, Bruker, Germany). Powder X-ray diffraction (PXRD) analysis was performed at 298 K using a PANalytical X’Pert PRO diffractometer (λ (Cu Kα) = 1.5418 Å) on a mounted bracket sample stage to compare the crystallinity of unloaded and drug-loaded NMOF. Data were obtained over the range of 5–65 °C. Thermal analysis was performed by LABSYS evo TGA STA DTA DSC by Setaram A trademark of KEP Technologies group. Experimental measurements were recorded from 20 to 800 °C under an air atmosphere with a heating rate of 20 °C/min. Transmission electron microscopy (TEM) (CEM 902A; Carl Zeiss, Oberkochen, Germany) was applied to examine the topography of selected NMOFs. Field emission scanning electron microscopy (FESEM, Zeiss Sigma 500 VP Analytical Carl Zeiss, Germany) was also carried out for the selected NMOF and NMOF-PEI-GA core–shell nanoparticles. The surface area, pore volume, and pore size distribution were analyzed by the Brunauer–Emmett–Teller (BET) method using a NOVA 2000e surface area and pore size analyzer (Quantachrome, Florida, FL) using nitrogen adsorption at 77 K in the range of 0.02 ≤ *P*/*P*_0_ ≤ 0.20.

#### 2.2.5 Determination of Drug Entrapment Efficiency (EE%) and Loading Capacity (LC%)

The entrapment efficiency (EE %) and loading capacity (LC %) of the loaded drug (DOX) into the DOX-loaded NMOFs were measured by the indirect method, where 50 mg of DOX was dissolved in 20 mL of deionized water and the concentration of DOX was measured by ultraviolet–visible (UV–vis) spectrophotometry by means of calibration curve at 480 nm, as described in the Supporting Information (Figure S1) and the Beer–Lambert plot for DOX. Afterward, 100 mg of activated NMOF was added to the DOX solution followed by sonication for 5 min and stirring for 72 h at room temperature. Finally, the solution was centrifuged, and the amount of the drug entrapped was detected spectrophotometrically at the wavelength λ_max_ = 480 nm.

All measurements were recorded three times. The DOX EE% was determined by applying the following equation
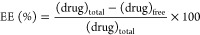
where (drug)_total_ is the total weight of DOX·HCl initially added to the NMOF aqueous suspension, while (drug)_free_ is the quantity of the nonentrapped free DOX·HCl in the supernatant.

The LC% was calculated using the following equation
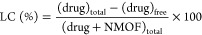
where (NMOF)_total_ is the total weight of the nanosized MOF initially added to the aqueous solution.

#### 2.2.6 In Vitro Drug Release

The in vitro release of free DOX and the DOX from DOX@NMOF-PEI-GA core–shell nanostructure was examined. A certain amount of DOX@NMOF-PEI-GA nanoparticles (1 mg/mL) was placed in a dialysis bag (MWCO = 3500) using phosphate buffer saline (PBS) buffer with different pH values (7.4, 6, 5.4, and 4). A shaker incubator with a shaking speed of 100 rpm at 37 °C was used for the release experiment. At preselected time intervals, 2 mL of the release medium was withdrawn and replaced with the same volume of fresh buffer. The amount of released drug was measured spectrophotometrically at a 480 nm wavelength. The measurements were replicated three times.

### 2.3 In Vitro Biological Assessments

#### 2.3.1 Biocompatibility and In Vitro Cytotoxicity

The biocompatibility of the DOX-free formulations NMOF-PEI and NMOF-PEI-GA was determined by testing several concentrations. Both hepatocellular carcinoma (HepG2) cells and the normal cells of human skin fibroblast (HSF) were used to determine the biocompatibility at 24 and 48 h, respectively. Additionally, the cytotoxicity assessment of the developed formulations of DOX@NMOF, DOX@NMOF-PEI, and DOX@NMOF-PEI-GA (at several variable ratios of DOX-NMOF to PEI-GA; 1:0.125, 1:0.25, and 1:0.5) in addition to the free DOX was performed using HepG2 cells.

HSF cells were kept in DMEM media treated with 100 mg/mL of streptomycin, 100 units/mL of penicillin, and 10% of heat-inactivated fetal bovine serum in a humidified atmosphere of CO_2_ at 37 °C. The sulforhodamine B (SRB) test (sulforhodamine B) was used to determine cell viability. Briefly, cell suspension aliquots (5 × 10^3^ cells) were distributed to the wells of a 96-well plate. Then, an additional 100 μL of the formula-treated media was added containing serial concentrations of NMOF-PEI-GA (10, 50, 100, 200, and 500 μg) to the cells’ suspension. After 3 days, the cell fixation was performed by removing the media and adding 150 μL of 10% TCA and incubated at 4 °C for 1 h. Then, the TCA was removed, and the cells were rinsed with distilled water 5 times. 70 μL of SRB (0.4% w/v) was added to each well and left for 1 h at a dark place for 10 min. Then, the wells were rinsed with 1% acetic acid 3 times and left to dry overnight. Then, 150 μL of 10 mM tris was added in order to solubilize the SRB. Finally, the absorbance measurement was collected using a BMG LABTECH-FLUOstar Omega microplate reader (Ortenberg, Germany) at 540 nm.

HepG2 cells were purchased from the American Type Culture Collection (ATCC, Rockville, MD). They were seeded in a 96-well tissue plate using RPMI-1640 medium supplemented with 10% of inactivated fetal calf serum and 50 μg/mL of gentamycin. Then, they were incubated in 5% of the CO_2_ atmosphere at 37 °C for subculturing 2–3 times weekly. In Corning 96-well tissue culture plates, the cell suspension concentration was 5 × 10^4^ cells/well in the growth medium and was incubated for 24 h before the treatment of the tested formulation. Then, several dilutions of the tested formulations (2, 7.8, 31.25, 125, 250, and 500 μg/mL) were added in triplicate to the wells. The controls were either the media or 5% DMSO for each plate. The population of viable cells was determined after incubation for 24 h using the MTT assay. Briefly, the media was replaced with a fresh one but lacking the phenol red and 10 μL of 12 mM MTT was added to all of the wells followed by a 4 h incubation in 5% of CO_2_ atmosphere at 37 °C. Then, 85 μL of the media was replaced by 50 μL of DMSO followed by 10 min of incubation at 37 °C. A microplate reader (SunRise, TECAN, Inc.) was used to determine the cell viability by recording the optical density at 590 nm. The IC_50_ was determined by plotting the responses to the formulations using GraphPad Prism software (San Diego, CA).

#### 2.3.2 In Vitro Assessment of Cellular Uptake by Immunofluorescence Microscopy

The in vitro cellular uptake of the developed nanostructures DOX@NMOF, DOX@NMOF-PEI, and DOX@NMOF-PEI-GA in addition to the free DOX was investigated by the immunofluorescence microscopy using HepG2 cells. HepG2 cells were cultured using RPMI-1640 culture (Gibco, ThermoScientific, Germany), treated with 10% fetal bovine serum (FBS) (Gibco, ThermoScientific, Germany) and 1% of penicillin G sodium salt (10,000 UI), streptomycin (10 mg), and amphotericin B (25 μg) (PSA) (Gibco, ThermoScientific, Germany). The cells were incubated for 24 h, and then the tested formulations were added using their IC_50_ values obtained at 24 h. The cellular uptake was examined by a LABOMED fluorescence microscope LX400, cat no: 9126000. Briefly, the cells were fixed with cold methanol and then stained with DAPI. The images were generated by OptikaI Sveiw software by applying 2 filters: the first one is the drug that emits a red color, and the excitation/emission were 470/595, respectively, and the second filter was DAPI, which emits a blue color, and the excitation/emission were 340/452, respectively. Violet color was produced upon the intersection of the red and blue colors.

#### 2.3.3 Cellular Apoptosis

In order to investigate the cellular apoptosis induced by the developed NOMOFs and analyze the cell cycle distribution, the flow cytometric analysis was carried out using HepG2 cells. The cells were incubated with DOX@NMOF-PEI and DOX@NMOF-PEI-GA in addition to the free DOX for 24 h. Then, the cells were harvested and washed twice with the binding buffer and PBS. Afterward, the cells were collected, suspended in 100 μL of the binding buffer and 1 μL of FITC-Annexin V (Becton Dickinson BD PharmingenTM, Heidelberg, Germany), and incubated for 40 min at 4 °C. Then, a mixture of 150 μL of binding buffer and 1 μL of DAPI (Invitrogen, Life Technologies, Darmstadt, Germany) with a concentration of 1 μg/mL in PBS was used to suspend the cells. Finally, the cells were analyzed by the flow cytometer BD FACS Calibur (BD Biosciences, San Jose, CA). By adopting flow cytometer techniques, the cellular apoptosis and cell were investigated.

#### 2.3.4 Cell Cycle Analysis

To analyze the cell cycle distribution, cell cycle analysis was carried out by the CycleTESTTM PLUS DNA Reagent Kit (Becton Dickinson Immunocytometry Systems, San Jose, CA). After incubation of HepG2 cells with DOX@NMOF-PEI and DOX@NMOF-PEI-GA in addition to free DOX, the cells were stained with propidium iodide stain as recommended by the kit and then analyzed by the flow cytometer. Cell cycle distribution calculation was performed by CellQuest software (Becton Dickinson Immunocytometry Systems, San Jose, CA).

### 2.4 Statistical Analysis

Two-way analysis of variance (ANOVA) software was used for the statistical analysis. The calculations were operated by SigmaPlot software 11.0. Differences were *P* < 0.001 (extremely significant), *P* < 0.01 (highly significant), and *P* < 0.05 (statistically significant). It should be noted that the (mean ± SD) was employed to represent the data.

## 3 Results and Discussion

The current research study involved the fabrication, characterization, and biological evaluation of a newly developed core–shell nanostructures of DOX-loaded NMOF (DOX@NMOF) core coated with PEI ligated with GA targeting ligand via the formation of amide bonds (DOX@NMOF-PEI-GA). The core–shell nanostructures were developed for enhanced active targeting and efficient delivery of DOX for HCC treatment. The ligated PEI was linked electrostatically with the prepared DOX-loaded NMOF in the presence of an alginate layer as a cross-linker interconnecting the DOX-NMOF core with the PEI-GA shell. The newly developed DOX@NMOF-PEI-GA was physiochemically characterized and its efficiency to selectively and actively target the HCC cells was investigated by the cytotoxicity and cellular uptake assays.

### 3.1 Physicochemical Characterization of PEI-GA and the Core–Shell Nanostructures, NMOF Nanostructures, NMOF-PEI and NMOF-PEI-GA, and the Shell PEI-GA

#### 3.1.1 Synthesis and Characterization of PEI-GA

**Figure 1:**
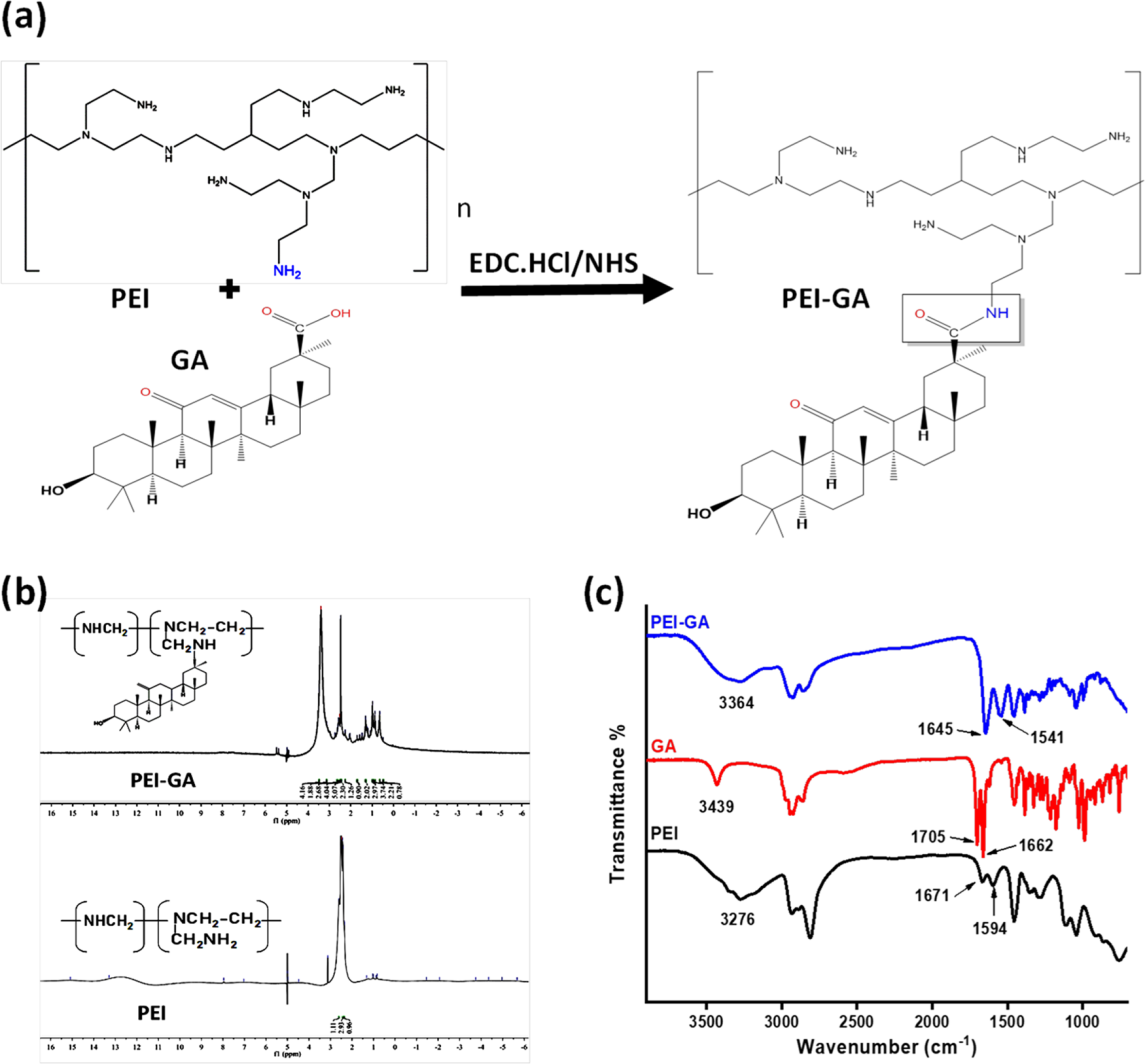
(a) Schematic representation of the synthesis of the PEI-GA conjugate and the characterizations of PEI-GA, GA, and PEI; (b) ^^1^^H NMR in *d*_6_-DMSO for PEI and PEI-GA; and (c) FTIR for PEI, GA, and PEI-GA.

The GA was conjugated to PEI with a (1:4) molar ratio through the formation of an amide bond between the GA carboxylic group and the amine groups of PEI by EDC/NHS chemistry (Figure 1a). PEI-GA formation was confirmed using ^1^H NMR and FTIR, as shown in Figure 1b,c, respectively. The ^1^H NMR spectrum indicated that the main three proton peaks of PEI (−NHCH_2_CH_2_−) were detected at 2.1–3.0 ppm, which agreed with previous reports.^49^ Conversely, when GA was conjugated to PEI, a new GA proton (aliphatic) peak appeared in the region 0.3–1.8 ppm, confirming the successful conjugation of GA onto the PEI backbone. Besides, in the PEI-GA conjugate, the main PEI three proton peaks were partially shifted downfield (3.0–4.0 ppm) due to the new neighboring amide group. Additionally, the FTIR spectrum of the GA-PEI conjugate (Figure 1c) displayed stretching peaks at 1645 and 1541 cm^–1^, which are attributed to −CO–NH– that further confirm the successful formation of the PEI-GA.

#### 3.1.2 Synthesis and Characterization of NMOF Core and NMOF-PEI and NMOF-PEI-GA Core–Shell Structures

**Figure 2:**
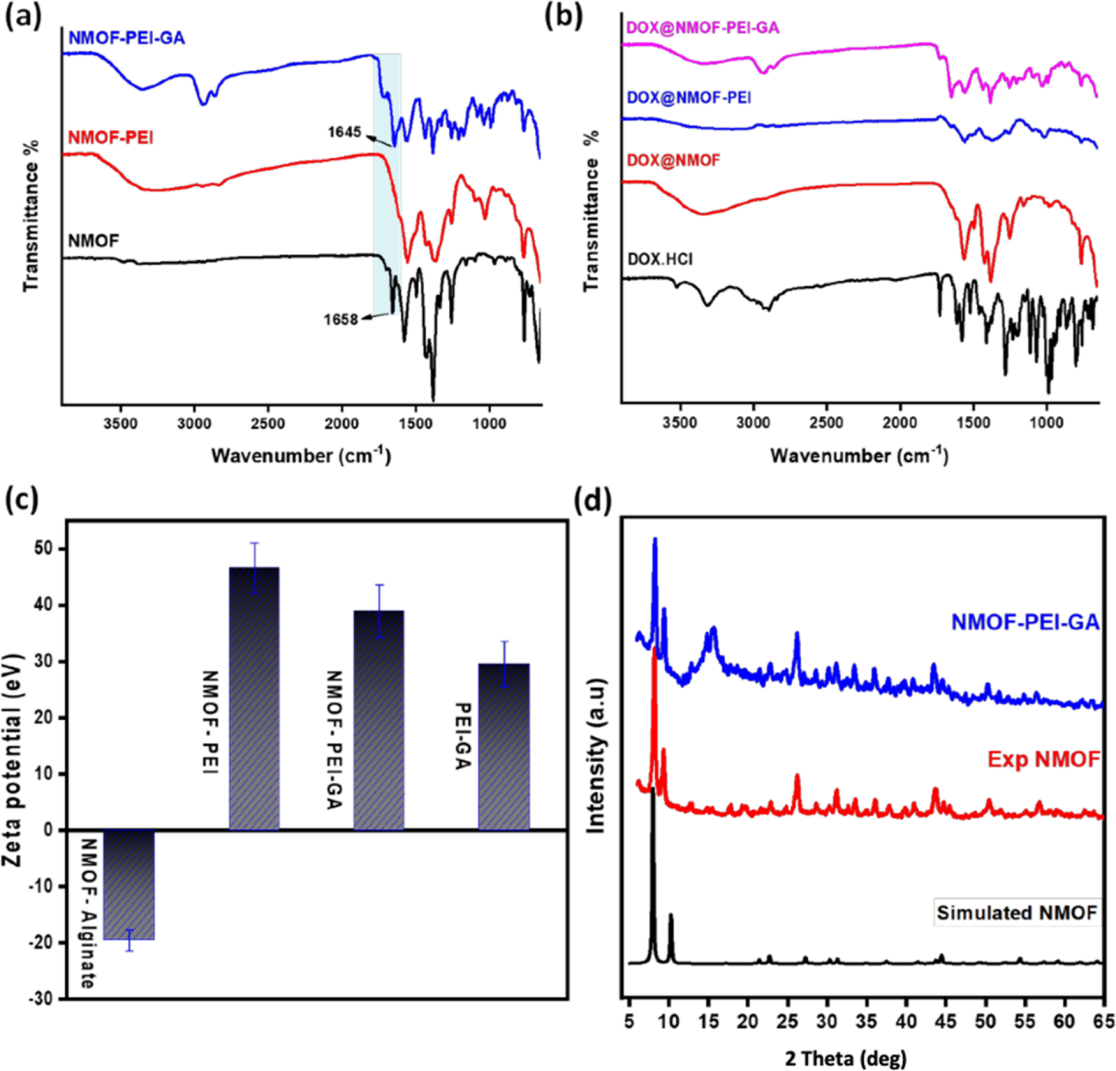
(a) FTIR spectra of NMOF, NMOF-PEI, and NMOF-PEI-GA; (b) FTIR spectra of DOX and DOX-loaded nanoformulations; (c) ζ-potential values for NMOF-alginate, NMOF-PEI, NMOF-PEI-GA, and PEI-GA; and (d) PXRD of the simulated NMOF, experimental NMOF, and the NMOF-PEI-GA.

**Scheme 1:**
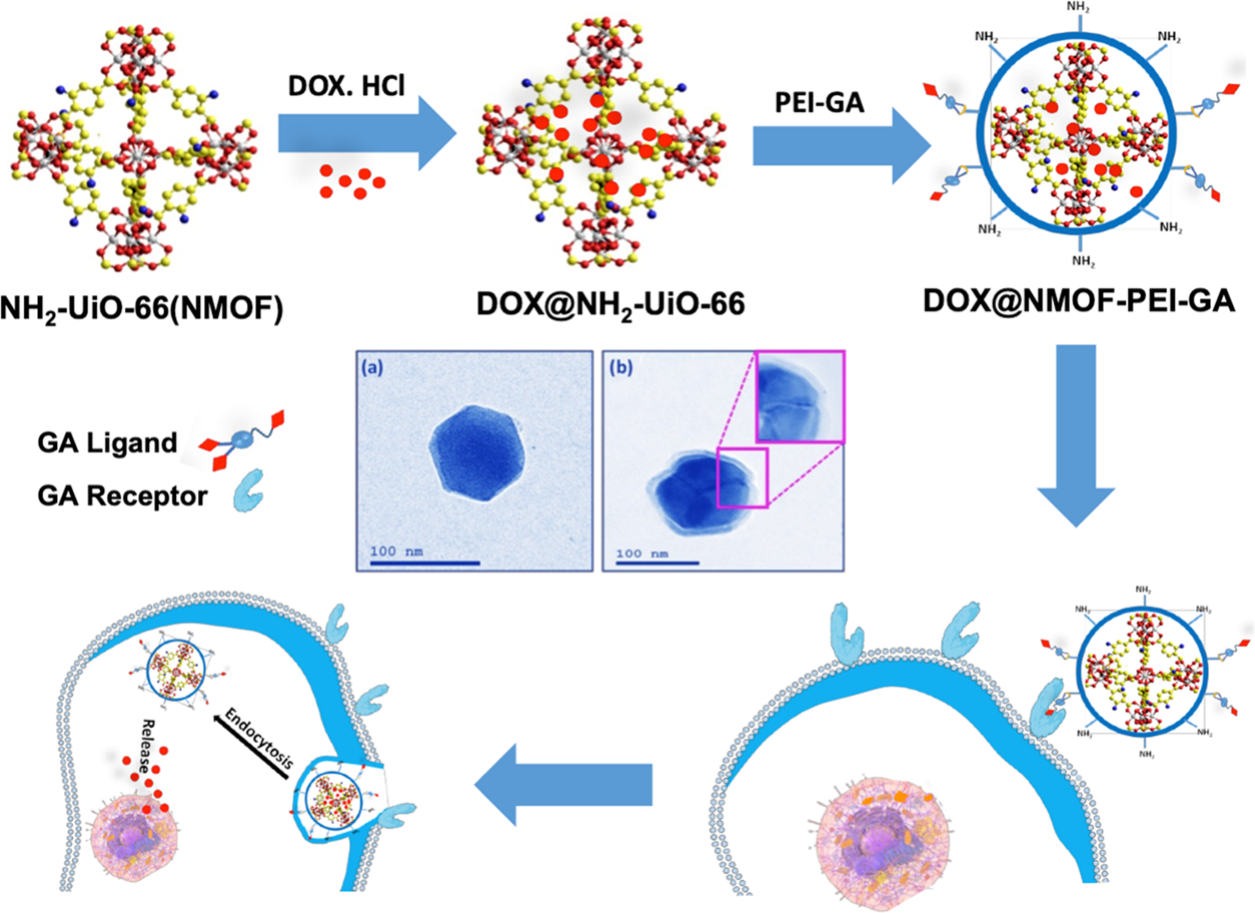
Systematic Presentation of the Developed Biocompatible Core–Shell Nanostructure Loaded with Doxorubicin (DOX) for Active Targeting of Hepatocellular Carcinoma (HCC), and TEM Micrographs for (a) NMOF and (b) NMOF-PEI-GA

The UiO-66-NH_2_ NMOF was fabricated according to the method reported previously.^48^ PEI or PEI-GA was then electrostatically linked to the NMOF in the presence of Na, the alginate (as an interconnecting cross-linking later) to coat the NMOF with the PEI-based shell, as shown in Scheme 1. The FTIR displayed in Figure 2 indicated some characteristics for NH_2_-UiO-66 NMOF and the modified NMOF. In the case of NH_2_-UiO-6, the appearance of a strong band at 1658 cm^–1^ is assigned to the *v* (C=O) stretching of *N*,*N*-dimethylformamide (DMF). However, the disappearance of this peak at NMOF-PEI proved the complete replacement of DMF with water.^50^ Furthermore, the stretching peak at 1645 cm^–1^ confirms the conjugation of GA to the PEI on the surface of NMOF. Instead, the DOX loading did not change the nanoformulation structure, which confirms the physical loading of drug into the nanoformulations as indicated in Figure 2b. In Figure 2c, ζ-potential revealed the surface charge of the NMOF before and after coating with either PEI or PEI-GA to form the core–shell nanostructures in an acidic aqueous medium mimicking the cancer acidic environment. NH_2_-UiO-66 NMOF depicted a positive charge on its surface, open metal sites, and the hydrophobic channel due to the Zr–OH on the Zr_6_ node forms Zr–OH_2_^+^ in aqueous solution at a pH environment below a value of 8.3.^17^ The alginate is an anionic polymer that would electrostatically bind to the cationic NH_2_-UiO-66 as well as create a cross-link between both the positively charged PEI and NH_2_-UiO-66.

In order to investigate the crystallinity of the as-synthesized NH_2_-UiO-66 and postmodified NMOF-PEI-GA, PXRD was applied as indicated in Figure 2d. NH_2_-UiO-66 presented narrow diffraction peaks in agreement with the simulated and previously reported data.^51^ The NMOF-PEI-GA was identical to the main peaks of the parent NH_2_-UiO-66, confirming that NH_2_-UiO-66 NMOF crystallinity was well maintained upon postmodification with the PEI-GA shell. However, the intensity of the NH_2_-UiO-66 peaks decreased after modification with PEI-GA, as well as a diffraction peak at 2Θ = 14 was noted, which is a good match to that of pure sodium alginate.^52^

#### 3.1.3 Thermal Stability and Surface Area Characterization

**Figure 3:**
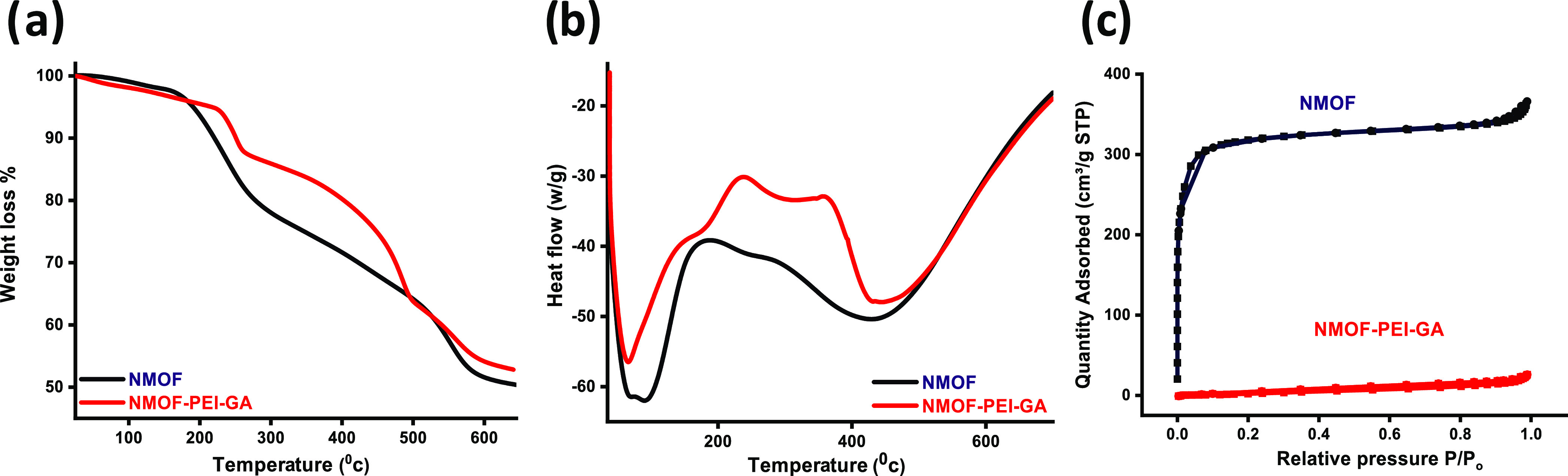
(a) Thermal analysis records for NMOF and NMOF-PEI-GA: (a) TGA, (b) DSC, and (c) N_2_ adsorption and desorption isotherm at 77 k for NMOF and NMOF-PEI-GA.

The thermogravimetric analysis (TGA) profiles for NMOF and the core–shell NMOF-PEI-GA are shown in Figure 3a, with all data recorded under a N_2_ gas. The thermal behavior of NMOF demonstrated a two-step weight loss. The weight loss started at 180–280 °C was assigned to the dehydroxylation of the zirconium oxo-clusters and removal of the adsorbed gas. The second weight loss (over 300 °C) was due to the decomposition of the framework and ligand. While the structure of the NMOF-PEI-GA thermogram displayed a higher thermal stability than the pristine NMOF, which was preserved up to 230 °C.

This enhancement proves the immobilization of PEI on the surface of the NMOF. Moreover, the DSC measurements for the NMOF and NMOF-PEI-GA illuminated the distinctive thermal behavior of the NMOF-PEI-GA as compared to that of the pristine NMOF, as shown in Figure 3b. The N_2_ adsorption and desorption isotherms at 77 K of the NMOF and NMOF-PEI-GA are illustrated in Figure 3c. The estimated BET surface area value for the pristine NMOF is 1145 m^2^/g, while the modified NMOF surface area dropped down to be 165 m^2^/g. The calculated data revealed a remarkable reduction in the NMOF-PEI-GA surface area that could be attributed to the low pore filling effect of N_2_ on the modified MOF surface, which caused blocking of the macropores between the MOF particles. This is a further confirmation of the formation of a core–shell nanostructure on the NMOF cluster via utilizing the alginate as a chemical cross-linker to bind the PEI on the NMOF surface.

#### 3.1.4 Surface Morphology and Topography Characterization

**Figure 4:**
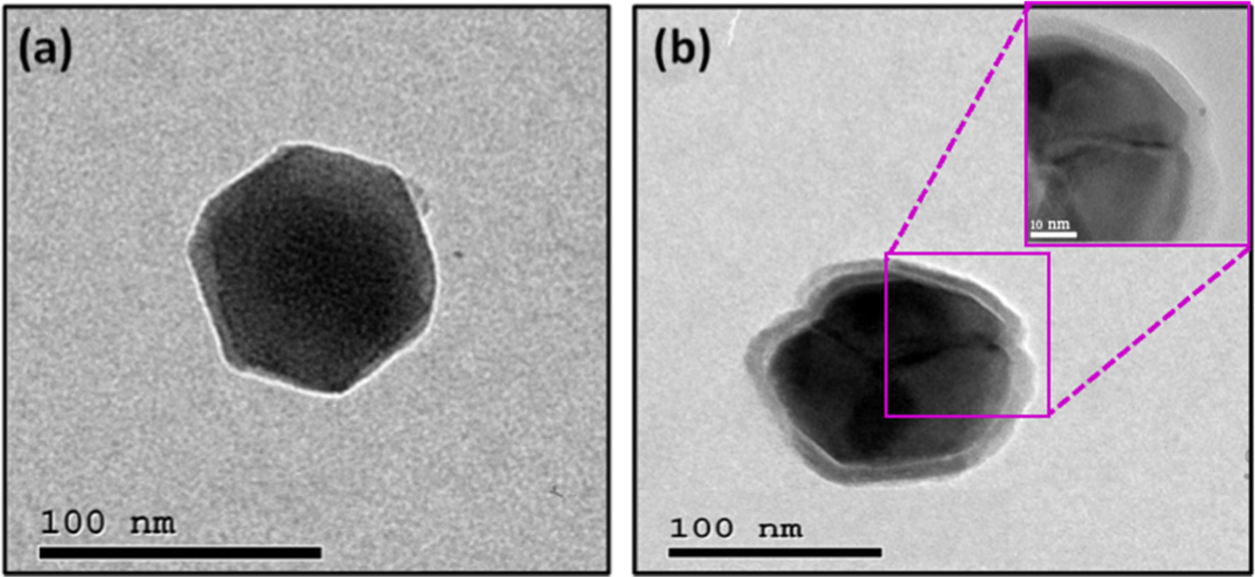
TEM micrographs for (a) NMOF and (b) NMOF-PEI-GA with the inset magnification to show the nanoshell formation.

To examine the morphology of synthesized nanoformulations, TEM was applied to investigate the microstructure of NH_2_-UiO-66 NMOF (core) and NMOF-PEI-GA (core–shell). As shown in Figure 4, the TEM micrographs clearly indicated the structure of NH_2_-UiO-66 NMOF as a hexagonal nanostructure with an average diameter of about 90 nm (Figure 4a). Additionally, Figure 4b shows the core–shell nanostructure (NMOF-PEI-GA) where NMOF is the core and PEI-GA is the shell.

Furthermore, the inset image in Figure 4b demonstrated a detailed view of the presence of the PEI-GA as a thin layer on the surface of NMOF. To further understand the effect of the PEI-GA modification on the morphology of NMOF, HR SEM measurements were conducted. Also, the energy-dispersive X-ray spectroscopy (EDX) was used to validate the porous NMOF (Figure S2). In addition, elemental mapping for the core–shell nanostructure (NMOF-PEI-GA) has been done to further confirm coating of the NMOF core with the ligated PEI, as indicated in Table S1. In addition, XPS (X-ray photoelectron spectroscopy) was performed to analyze the surface composition of NMOF-PEI-GA. In Figure S3, the XPS detected the presence of C, N, and O. The N 1s spectrum, shown in Figure 3c, exhibited two distinct peaks at 399.4 and 400.1 eV, which corresponded to N–C amide bonds and protonated amine groups, respectively. These findings are consistent with previous research^53^ and suggest that the targeting ligand GA was successfully linked to the PEI.

### 3.2 Encapsulation Efficiency Studies

The obtained results showed that the porous nanostructure of the NMOF allowed a high loading efficiency of the drug molecules. For instance, DOX was encapsulated into the NMOF porous structure with high drug payload, where the attained EE and LC% were 70 and 23%, respectively. The DOX loading step was performed after the NMOF synthesis and prior to PEI-GA coating. The obtained results are in good agreement with the previous studies that revealed that the sufficient encapsulation of the drug inside the NMOF pores may be hampered by its hydrophilic characteristics.^54^ Additionally, the polymer (alginate and PEI) coating the NMOF is a critical factor in both effective drug encapsulation and drug release from the nanocarrier. The EE% of the drug in a nanocarrier relays particularly on the drug solubility in the polymer solution.^17^ Owing to the nonvolatile nature of DOX, its EE% into the core–shell nanostructure (DOX@NMOF-PEI-GA) was measured by UV spectrophotometry and found to be about 97%. The developed NMOF can hold the drug or other small biological molecules within its pores and channels. Furthermore, coating the NMOF with a polymer allows good stability for the drug-loaded NMOF system. Nevertheless, when the nanostructure is exposed to different pH values and times, the coating polymer decomposes gradually and the drug is released.^55^

### 3.3 In Vitro Release Study

**Figure 5:**
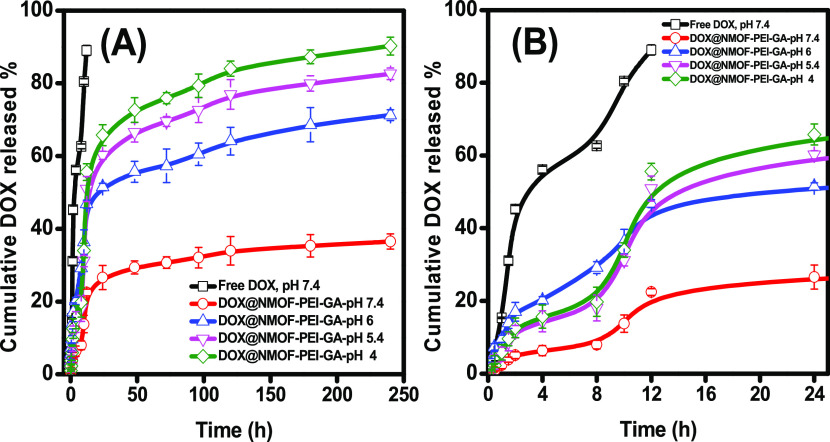
pH-dependent cumulative release profiles of free DOX and DOX from the developed DOX@NMOF-PEI-GA core–shell nanostructures for 240 h (A) and within the first 24 h (B) in PBS at pH values 7.4, 6.0, 5.4, and 4.

The in vitro drug release in the cancer microenvironment could be controlled by changing the pH owing to oxygen and nutrition deficiency.^56^ The endothelial tumor cell pH value is near 5, and the neutral physiological condition is around 7.4.^57^ The in vitro cumulative (pH-dependent) release of the loaded DOX from the developed DOX@NMOF-PEI-GA nanocarriers was investigated at different pH values. From the results in Figure 5, it can be noted that the free DOX passed rapidly through the dialysis bag, while the DOX loaded into the nanostructure DOX@NMOF-PEI-GA was retained and released in a sustained manner at all of the tested pH values with different rates. To investigate the effect of changing the pH on the drug release behavior of NMOF-PEI-GA, equivalent quantities of DOX@NMOF-PEI-GA were injected into PBS solutions of four different pH values in dialysis bags, and at predetermined time intervals, the PBS was withdrawn and replaced by a fresh buffer. Then, the DOX concentration outside the bag was measured spectrophotometrically at the corresponding absorbance wavelength of the DOX (480 nm).

Figure 5 indicates the amount of DOX release pattern of either the free DOX at pH 7.4 or from DOX from DOX@NMOF-PEI-GA at different pH values (4, 5.4, 6, and 7.4). By changing the pH from 7.4 to 4, the amount of the released DOX increased significantly with the maximum amount released at pH 4. These results indicate that the developed NMOF-PEI-GA nanocarriers were able to release the DOX in the acidic tumor microenvironment. Furthermore, the free DOX was totally released after the first 12 h, while the DOX release from the DOX@NMOF-PEI-GA core–shell nanocarriers conferred a sustained release of DOX for more than 4 days at acidic pH values (4, 5.4, and 6).

At pH 4, the cumulative release of DOX attained 65, 72, and 76% after 24, 48, and 72 h, respectively. Nevertheless, only 30% was released at pH 7.4 after 72 h. The fast release of DOX under an acidic physiological environment might be due to the protonation of the amine groups of PEI, which leads to the repulsion of the charged molecules and their swelling under acidic pH.^47^ Consequently, the PEI shell would be less compact and allow the leakage of the PBS buffer to reach the DOX@NMOF core and thus liberating the drug. Also, the physically entrapped DOX into the NMOF without any chemical conjugation would enhance the drug’s release rate. The obtained results validated the high efficiency of the designed nanodrug delivery system (DOX@NMOF-PEI-GA) in prolonging the release time of the DOX from it. This might be explained by the strong hydrophobic interaction between DOX and GA and in a good match with the previously reported study.^39^

### 3.4 In Vitro Biological Assays

#### 3.4.1 Biocompatibility and Cytotoxicity Assays

**Figure 6:**
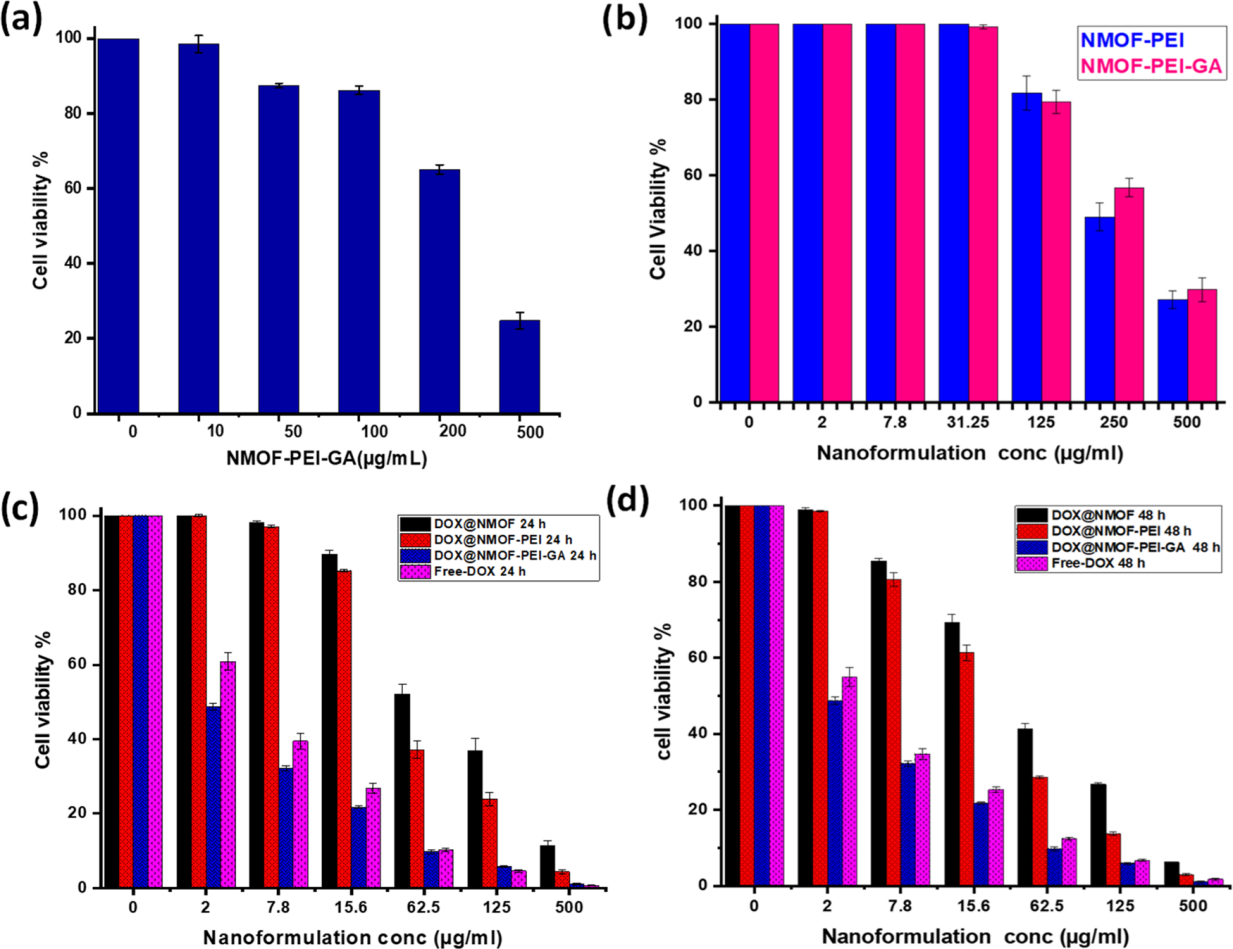
(a) Biocompatibility assessment performed by DOX-free NMOF-PEI-GA-treated HSF cells for 48 h, (b) biocompatibility assessment of HepG2 cells after treatment by the DOX-free formulations NMOF-PEI and NMOF-PEI-GA for 24 h, and (c, d) cytotoxicity assay after incubating the HepG2 cells with the developed nanoformulations in addition to the free DOX at 24 and 48 h.

The biocompatibility of the DOX-free formulations NMOF-PEI and NMOF-PEI-GA was tested on both types of cells; HSF and HepG2 cells as representatives for the normal and HCC tissues, respectively, in order to investigate any carrier-related cytotoxicity. The biocompatibility of the DOX-free NMOF-PEI-GA was determined by incubating serial concentrations (10, 50, 100, 200, and 500 μg) with HSF cells for 72 h (Figure 6a). As shown in the results, about 90% of the cells’ population remained viable up to a concentration of 100 μg/mL, and the viability began to decline at higher concentrations of the formulation. Consequently, the NMOF-PEI-GA is considered a biologically safe and biocompatible drug carrier fulfilling the requisites of the drug delivery systems.

The biocompatibility of NMOF-PEI and NMOF-PEI-GA was assessed using the HepG2 cell using the concentrations of 2, 7.8, 31.25, 125, 250, and 500 μg/mL. As shown in Figure 6b, both the two formulations NMOF-PEI and NMOF-PEI-GA exhibited minimal cytotoxicity toward the HepG2 cells at a concentration of 125 μg/mL, where about 80% of the cells remained viable. Additionally, these results prove that the GA ligation to the cationic polymers as general and to PEI specifically has partially consumed and neutralized the PEI positive charge. Thus, the toxicity of the PEI greatly declined and rendered the nanoformulations safe and biocompatible.^58^ The biocompatibility results are in great accordance with what was previously reported regarding PEI toxicity. Consequently, the IC_50_ values of both the drug-free formulations NMOF-PEI and NMOF-PEI-GA indicated that they are biocompatible nanocarriers for active drug delivery.

The DOX was used as a model anticancer drug due to its superior effectiveness against several cancer types such as HCC.^30,43,44^ Several ratios of DOX@NMOF and PEI-GA were used to achieve the optimum one (details in Table S2). Intriguingly, DOX@NMOF-PEI-GA with a ratio of 1:0.5 w/w of DOX@NMOF to PEI-GA had the most potent IC_50_ (6.89 ± 0.38 μg/mL), so it is the most efficient formulation for the enhanced selective and active drug delivery of the DOX to the HepG2 cells. Consequently, a ratio of 1:0.5 w/w was chosen to complete further assessments.

In an attempt to determine the hepatocellular toxicity of the developed nanoformulations, the free DOX in addition to DOX@NMOF, DOX@NMOF-PEI, and DOX@NMOF-PEI-GA was used to treat HepG2 cells and the IC_50_ values at 24 and 48 h were determined using the MTT assay. The enhanced effect of DOX@NMOF-PEI-GA (IC_50_ = 6.89 ± 0.38 and 1.94 ± 0.12 μg/mL at 24 and 48 h, respectively) compared to that of DOX@NMOF (IC_50_ = 71.4 ± 2.78 and 39.23 ± 1.59 μg/mL at 24 and 48 h, respectively, *p* = 0.0001) or DOX@NMOF-PEI (IC_50_ = 47.55 ± 2.91 and 25.2 ± 1.28 μg/mL at 24 and 48 h, respectively, *p* = 0.0001) or the free DOX (5.88 ± 1.42 and 2.81 ± 0.37 μg/mL at 24 and 48 h, respectively *p* = 0.0098 and 0.0005) (Figure 6c,d) can be elicited. These results emphasize the role of both the PEI and GA in enhancing the active drug delivery of DOX to the HCC cell. PEI has been previously reported to enhance endocytosis and transfection by escaping the endosomes, thereby facilitating cellular entry and intracellular cargo delivery.^38^ Herein, the PEI enhanced the endocytosis of the novel nanoformulation, which is the DOX-@ NMOF-PEI-GA and thus facilitated the intracellular drug delivery. Due to the existence of GA receptors on the HCC cells’ surface, GA was reported to decorate the nanocarrier surface to achieve active drug delivery to HCC.^35,36,59^ Consequently, the selectivity of the nanoformulations toward the HepG2 cells was fortified by the ligation of the GA to the PEI surface. The enhanced selectivity resulted in increasing the DOX amount delivered intracellularly and then to the nucleus as the drug is carried in the pores of the NMOF, which is coated by the PEI-GA. This enhancement is evidenced by the decline in the IC_50_ values at both time intervals 24 and 48 h; all of the cytotoxicity data are mentioned in Table S3.

#### 3.4.2 In Vitro Assessment of Drug Cellular Uptake by Fluorescence Microscopy

**Figure 7:**
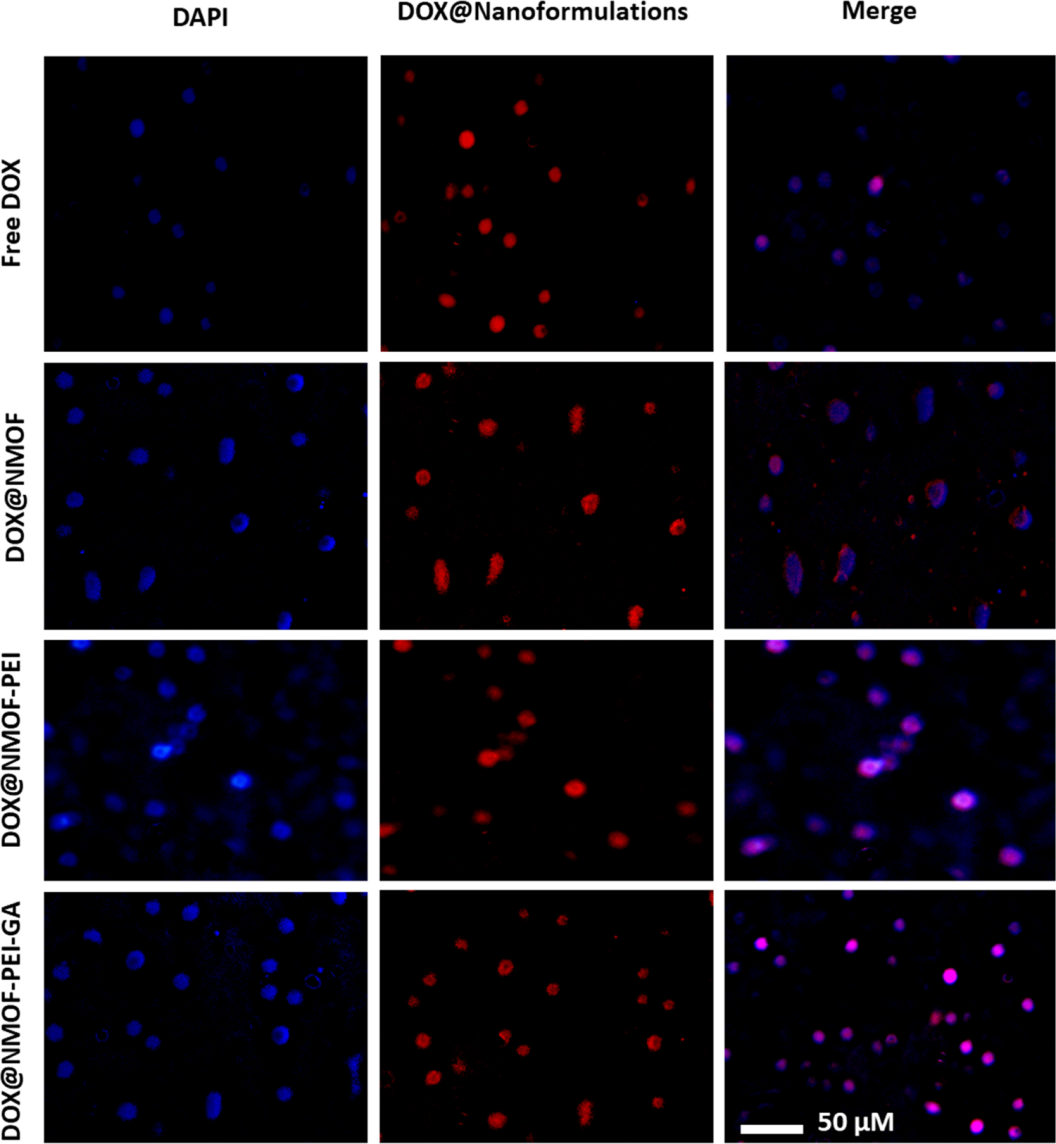
Immunofluorescent images for HepG2 cells treated with three doxorubicin-loaded nanoformulations and free DOX for 24 h. The images were captured with a LABOMED fluorescence microscope LX400, cat no: 9126000. The magnification power is 10×.

To visualize the cellular uptake and identify the intracellular localization, DOX@NMOF, DOX@NMOF-PEI, and DOX@NMOF-PEI-GA as well as free DOX were tested on HepG2 cells at 24 h using the fluorescence microscopy (Figure 7). The emerging red fluorescence confirms the intranuclear localization of the DOX-loaded formulations. These results were accentuated by the emergence of violet fluorescence, which is obtained from the nuclear localization (blue fluorescence) and DOX localization inside the nucleus (red fluorescence).

It is worth noting that DOX@NMOF-PEI-GA had superior nuclear localization when compared to the other tested nanoformulations, which may be attributed to enhanced cellular targeting and internalization. These results are in accordance with the cytotoxicity results. The nuclear delivery of the DOX by the DOX@NMOF-PEI-GA nanostructure may also be attributed to the ability of the PEI to escape the endosomal/lysosomal uptake and reach the cytoplasm.^60^ Then, the PEI would reach the nuclear membrane without accumulation inside the nuclei where it creates local holes either by tiny disruption of the membrane or by PEI-supported holes.^60^ Through these holes, the PEI delivers its cargo^61^ and, in this study, it delivered the DOX to the nuclei. Consequently, the results revealed a cellular-enhanced active targeting of the HCC cells through the GA–GA receptor binding and subcellular (nuclear) targeting by the PEI-based shell to deliver the DOX to the nucleus.

#### 3.4.3 Flow Cytometry Analysis Results

In order to validate the effect of the developed nanosystems on cellular apoptosis, flow cytometry analysis of the samples free DOX, DOX@NMOF, and DOX@NMOF-PEI-GA were carried out using the IC_50_ at 24 h.

**Figure 8:**
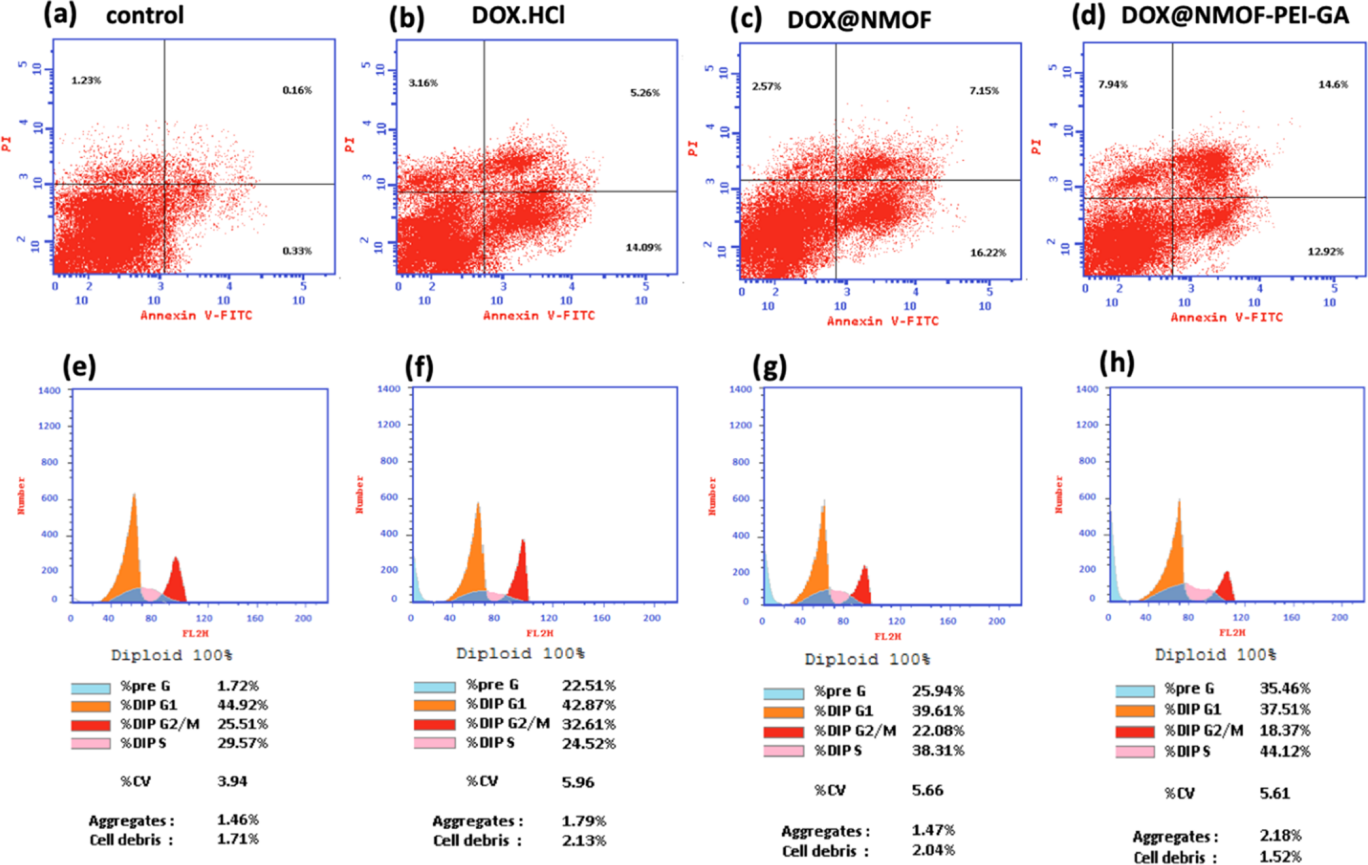
Histograms of flow cytometry analysis of HegG2 cells: (a) negative control (untreated cells), (b) treated with free DOX, (c) treated with DOX@NMOF, and (d) treated with DOX@NMOF-PEI-GA. Cell cycle analysis of the DNA content of HepG2 cells: (e) control cells, (f) treated with free DOX, (g) treated with DOX@MOF, and (h) treated with DOX@MOF-PEI-GA. IC_50_ at 24 h was used.

Consequently, HepG2 cells were treated with the samples, while the untreated cells represented the negative control (Figure 8a–d). The cells were classified into three subpopulations. The first one is the live cells, which were stained neither by annexin V (AV) nor by propidium iodide (PI) (AV–/PI−). The second one represents the early apoptotic cells, which are (AV+/PI−) due to the presence of many phosphatidyl cholines. The last population is both the late apoptotic and necrotic cells (AV–/PI^+^). From the results represented in Table S4, it can be concluded that both the DOX@MOF and DOX@MOF-PEI-GA significantly reduced the live cells (77.49 and 64.54%, respectively) in the HepG2 cells when compared to the control (98.28%). Additionally, DOX@NMOF-PEI-GA had significantly increased the apoptosis of the cells (27.52%) more than DOX@NMOF (19.35%) (*p* = 0.0009 and <0.0001 for the early and late apoptosis, respectively), the free DOX (23.37%) (*p* = <0.0001 for both early and late apoptosis), and the control cells (0.49%) (*p* = <0.0001 for early and late apoptosis, respectively), which evidence the fortified effect and selectivity of the DOX@NMOF-PEI-GA to actively deliver the DOX. The enhanced apoptosis of the developed nanoformulation DOX@NMOF-PEI-GA (especially the late apoptosis) is attributed to the reported effect of the GA to enhance the apoptosis.^62^ Consequently, the addition of the PEI-GA to the DOX@NMOF increased the late apoptosis from 5.26 to 14.6%, and these results are in concordance with the literature. Finally, the DOX@NMOF-PEI-GA had a higher necrotic effect on the cells (7.94%) when compared to the DOX@NMOF (3.16%) (*p* = <0.0001) and the free DOX (2.57%) (*p* = <0.0001), which emphasize the significant role of the novel nanoformulation in targeting and killing the hepatic cancer cells. The enhanced necrotic effect of DOX@NMOF-PEI-GA as compared to that of DOX@NMOF can be attributed to the GA-induced necrosis, which is due to the GA-induced lactate dehydrogenase intracellular release as previously reported in the literature.

The flow cytometry analysis of the DNA content was also performed to investigate the cell cycle kinetics using the 24 h IC_50_ of the DOX@NMOF, DOX@NMOF-PEI-GA, and the free DOX in addition to the untreated HepG2 cells as a negative control (Figure 8e–h); all data values are mentioned in Table S5. From the obtained results, both the DOX@NMOF-PEI-GA and free DOX decreased the cell population in the G0/G1 phase (37.51 and 39.61%, respectively), while the DOX@NMOF slightly decreased the cell population (42.87%) compared to the control cells (44.92%).

Additionally, DOX@NMOF-PEI-GA enhanced the cell death at the S-phase by 1.49-fold compared to the control. Also, the results demonstrated that the cycle growth arrest of DOX@NMOF-PEI-GA in the G2/M phase was decreased to 72% compared to the control cells. Finally, the DOX-loaded nanoformulations significantly changed the pre-G1 phase by increasing the cell populations and the DOX@NMOF-PEI-GA showed the highest increase by about 20-fold, while the DOX@NMOF and the free DOX increased by 13- and 15-fold, respectively, compared to the control. These results provide evidence to the previously illustrated flow cytometry histogram results that demonstrated an enhanced cell death by the developed novel nanoformulations at the apoptotic stage.

## 4 Conclusions

In conclusion, a biocompatible and highly efficient nano-DDS was successfully designed and developed. The developed nano-DDS was composed of a Zr-based NMOF (NH_2_-UiO-66) core with the tumor-targeting glycyrrhetinic acid (GA) ligated to polyethyleneimine (PEI) as the shell, and doxorubicin (DOX) was loaded as a model anticancer drug. GA was chemically conjugated to PEI to improve the targeting activity toward HCC and to reduce PEI cytotoxicity. The developed free/DOX-loaded nanosystems were subjected to various physicochemical characterizations including electron microscopy, XRD, H NMR, and in vitro evaluation (e.g., biocompatibility, antitumor activity, and cellular uptake), revealing the successful formation and the efficiency of the developed core–shell nanostructure. The developed nano-DDS showed significant results such as (a) high loading and encapsulation efficiency, (b) minimal toxicity toward both normal human skin fibroblast (HSF) and hepatic carcinogenic (HepG2) cells, (c) controlled drug release, (d) pH-simulated response, and (e) enhanced drug-selective properties and a superior killing effect toward hepatic tumor cells. Besides, the fluorescence imaging of HepG2 cells treated with the developed DOX-loaded NMOF showed clear localization of the drug inside the cells. In summary, the core–shell NMOF-PEI-GA nanocarrier developed in the present study may offer a promising nanoplatform for targeted hepatic carcinoma therapy after further preclinical and clinical evaluations and improvement.

## Data Availability

All data are available with the corresponding authors upon any reasonable request.

## Supporting Information Available

Synthesis of nanosized NH_2_-UiO-66 NMOF and loading with the anticancer DOX drug; calibration curve of DOX·HCl, HR SEM, and elemental mapping of NMOF and NMOF-PEI-GA, XPS analysis of NMOF-PEI-GA, and tables of in vitro assessment results (PDF)
