# Supplementary material for: Core–Shell Nanostructured Drug Delivery Platform Based on Biocompatible Metal–Organic Framework-Ligated Polyethyleneimine for Targeted Hepatocellular Carcinoma Therapy
Source: ACS Omega. 2023 May 31;8(23):20779–91. doi: 10.1021/acsomega.3c01385 (PMC10269253; doi:10.1021/acsomega.3c01385)
Supplement: Supplementary file 1 — ao3c01385_si_001.pdf [file ao3c01385_si_001.pdf]

Supporting Information

Core-shell nanostructured drug delivery platform based on biocompatible metal organic framework-ligated polyethyleneimine for targeted hepatocellular carcinoma therapy.

Mostafa Fytory[1,2], Amira Mansour[1], Waleed M.A. El Rouby[2], Ahmed A. Farghali[2], Xiaorong Zhang[4], Frank Bier[4], Mahmoud Abdel-Hafiez[3*], and Ibrahim M. El-Sherbiny[1,*]

Authors information

Corresponding authors:

Ibrahim M. El-Sherbiny: *Nanomedicine Labs, Center for Materials Science (CMS), Zewail City of Science and Technology, 6 October City, 12578, Giza, Egypt.*
*Email: ielsherbiny@zewailcity.edu.eg

Mahmoud Abdel-Hafiez: *Department of Physics and Astronomy, Uppsala University, Box 516, SE-75120 Uppsala, Sweden.*
*Email: mahmoud.hafiez@physics.uu.se

Authors:

Mostafa Fytory: *Nanomedicine Labs, Center for Materials Science (CMS), Zewail City of Science and Technology, 6 October City, 12578, Giza, Egypt.*
Material Science and Nanotechnology Department, Faculty of Postgraduate Studies for Advanced Sciences (PSAS), Beni-Suef University, 62511 Beni-Suef, Egypt.
Amira Mansour: *Nanomedicine Labs, Center for Materials Science (CMS), Zewail City of Science and Technology, 6 October City, 12578, Giza, Egypt.*
Waleed M.A. El Rouby: *Material Science and Nanotechnology Department, Faculty of Postgraduate Studies for Advanced Sciences (PSAS), Beni-Suef University, 62511 Beni-Suef, Egypt.*
Ahmed A. Farghali: *Material Science and Nanotechnology Department, Faculty of Postgraduate Studies for Advanced Sciences (PSAS), Beni-Suef University, 62511 Beni-Suef, Egypt.*
Xiaorong Zhang: *Molecular Bioanalytics and Bioelectronics Group, Institute of Biochemistry and Biology, University of Potsdam, 14476 Potsdam-Golm, Germany.*
Frank Bier: *Molecular Bioanalytics and Bioelectronics Group, Institute of Biochemistry and Biology, University of Potsdam, 14476 Potsdam-Golm, Germany.*

Table of contents:

SI.1: Additional experimental details.

SI.2: HR SEM and elemental mapping of NMOF and NMOF-PEI-GA.

SI.3: XPS analysis of NMOF-PEI-GA.

SI.4: In vitro assessment by cytotoxicity and flow cytometry.

SI.1: Additional experimental details:

Synthesis of nanosized NH_2-UiO-66 NMOF and loading with the anticancer DOX drug.

Briefly, 125 mg of $ZrCl_4$ was dissolved in a mixture of DMF:HCl with a volume ratio of 10:1, and sonicated for 15 min. Afterward, a DMF solution containing 2-aminobenzenedicarboxylic acid (NH_2-BDC, 135 mg) was injected to the above solution and further sonicated for additional 15 min, then the mixture was heated at 80 °C for 24 h. The resulted powder was collected and washed several times with DMF before immersing in acetonitrile for solvent exchange. Finally, the yield was dried under vacuum at 60 °C overnight, and the obtained NMOF was activated at 150 °C for 4 h before further use.

DOX.HCl was physically loaded into the synthesized NMOF. Briefly, 100 mg of NMOF dispersed in 10 ml of deionized water was sonicated for 5 min. Afterwards, 10 ml of DOX solution (5 mg/ml) in distilled water was added and the mixture was stirred for 72 h. The loading experiment was performed under dark conditions in a sealed bottle, and left to stir at 450 rpm for 72 h. Then, the mixture was collected by centrifugation at 9000 rpm for 10 min.

The unentrapped drug was calculated by analyzing the supernatant by UV-Vis spectrophotometry at wavelength of 480 nm. Eventually, the DOX-loaded NMOF was washed with water and dried under vacuum at room temperature.

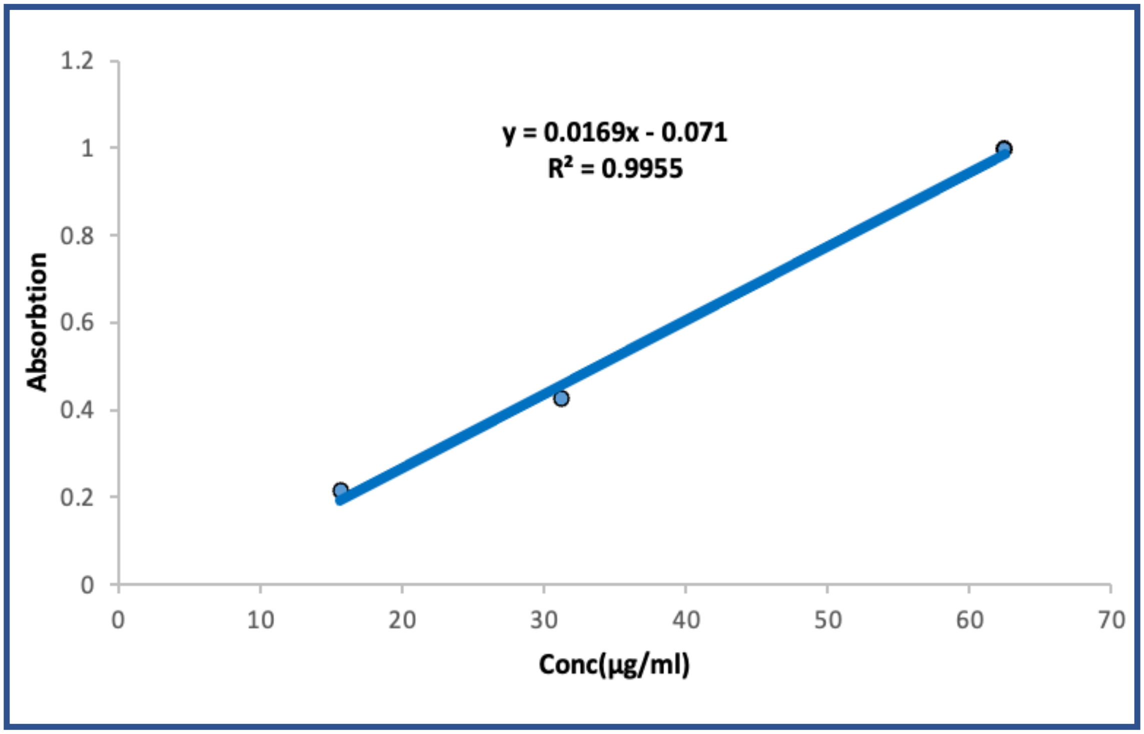

Figure S1: Calibration curve of DOX.HCl

SI.2: HR SEM and elemental mapping of NMOF and NMOF-PEI-GA

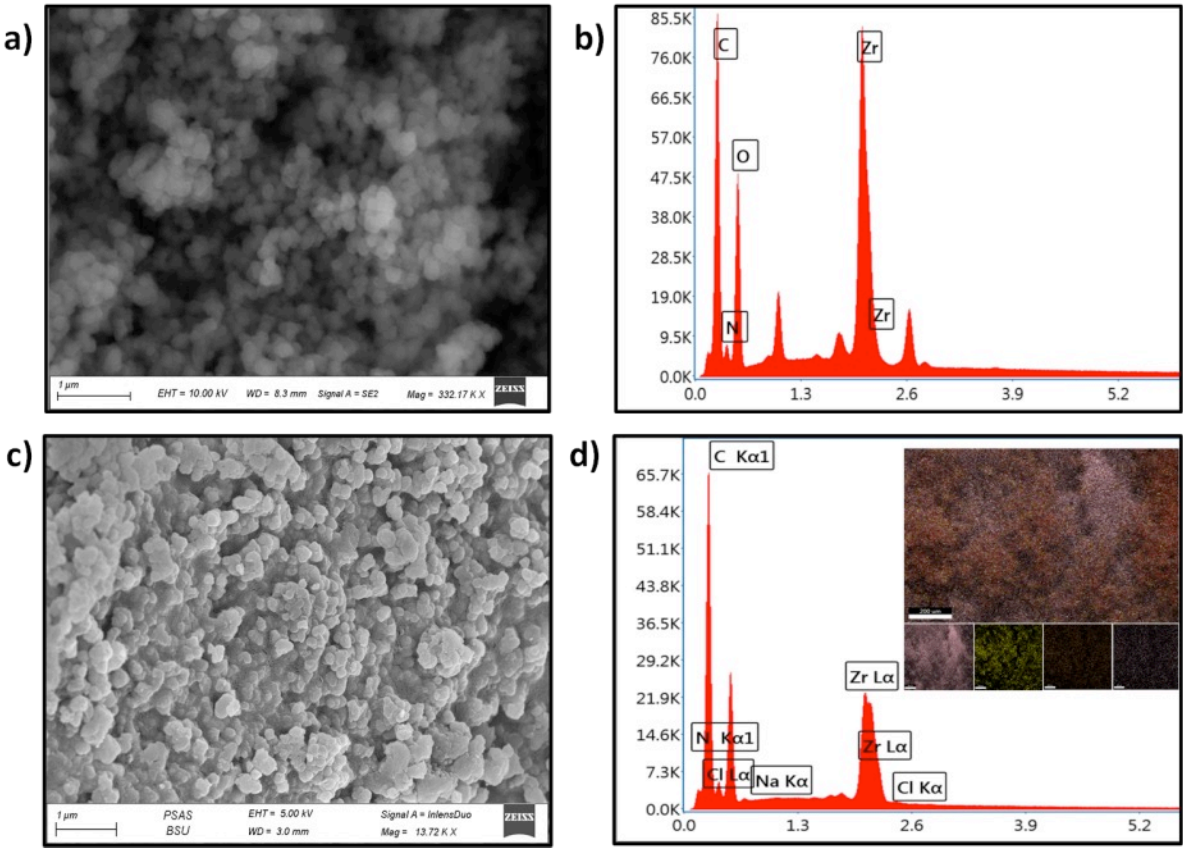

Figure S2 a-b. HR-SEM for NMOF, and EDX analysis for as-synthesized NMOF, respectively, **(c)** HR-SEM for the core-shell NMOF-PEI-GA, and **(d)** elemental mapping for NMOF-PEI-GA.

To further understand the impact of PEI-GA modification on the morphological structure of NMOF, HRSEM measurements were conducted which showed that NH_2-UiO-66 has a slightly spherical structure **Figure S2a, b**. **Figure S2c** shows that in the case of NMOF-PEI-GA, the surface and inter-crystal of NH_2-UiO-66 are completely covered and occupied by PEI-GA. support **Figure 5b**. In **Figure S2d**, the consistency of NMOF-PEI-GA was verified, where the Zr, Na and C maps were prominent, with the increase in appearance of nitrogen (N) that could be attributed to the amine groups of PEI and confirmed the coating of NMOF with PEI-GA as reported in **Table S1**.

Table S1: EDX and elemental mapping of the core shell nanostructure (NMOF-PEI-GA)

Element	Weight %	Atomic %	Net Int.	Error %	Kratio	Z	A	F
C K	60.94	73.34	823.43	7.29	0.2440	1.0509	0.3810	1.0000
N K	22.91	23.64	79.89	11.71	0.0201	1.0271	0.0855	1.0000
NaK	0.96	0.61	25.79	7.80	0.0044	0.9149	0.4979	1.0019
ZrL	15.18	2.41	380.95	2.71	0.1234	0.7067	1.1504	1.0000
ClK	0.01	0.00	0.30	78.70	0.0001	0.8545	0.8859	1.0039

SI.3: XPS analysis of NMOF-PEI-GA

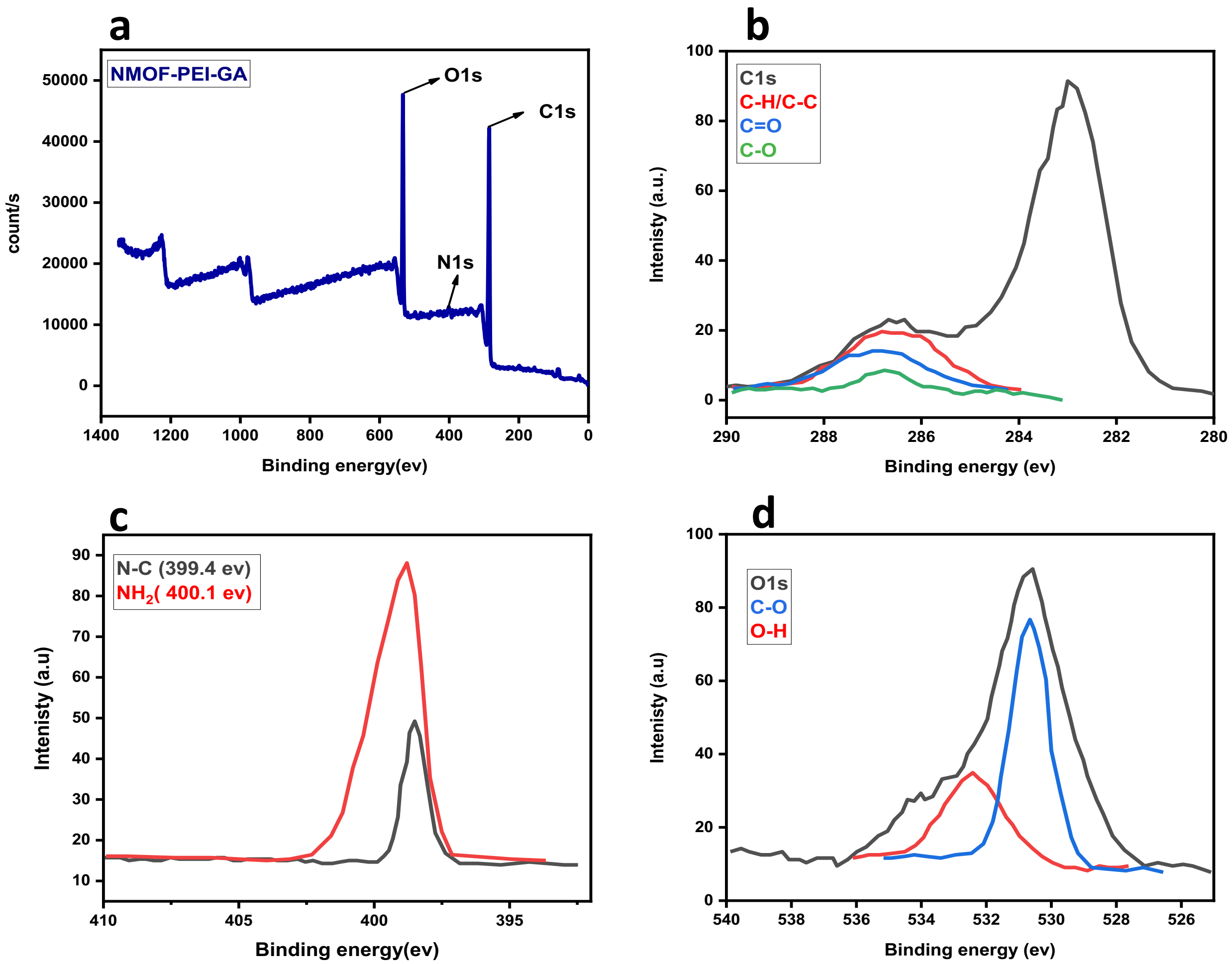

Figure S3 a-d: XPS analysis of the full region of NMOF-PEI-GA(a) and different elements C 1s (b), N 1s (c), and O 1s (d).

SI.4: In vitro assessment by cytotoxicity and flow cytometry

In order to determine the optimal PEI concentration, several ratios of DOX@NMOF to PEI-GA (1:0.125, 1:0.25 and 1:0.5) were investigated to identify the best formulation of DOX@NMOF-PEI-GA. DOX@NMOF-PEI-GA with the ratio of 1:0.5 wt/wt of DOX@NMOF to PEI-GA had the most potent IC_{50} (6.89 ± 0.38 µg/ml).

Table S2. Several ratios of DOX@NMOF to PEI-GA and their corresponding IC_{50} values at 24 h

DOX@NMOF: PEI-GA ratio (w/w)	IC_{50}/24 h (µg/ml) ± SD
1:0.125	23.45 ± 0.87
1:0.25	11.55 ± 0.89
1:0.5	6.89 ± 0.38

Table S3: Cytotoxicity of the free DOX in addition to the DOX-loaded NMOF formulations (DOX@NMOF, DOX@NMOF-PEI and DOX@NMOF-PEI-GA) at 24 and 48 h.

Sample	IC50/ 24 h (µg/ml) ± SD	DOX (µg) ± SD	IC50/ 48 h (µg/ml) ± SD	DOX (µg) ± SD
**DOX.HCl**	5.88 ± 1.42	--------	2.81 ± 0.37	5.88 ± 1.42
**DOX@NMOF**	71.4 ± 2.78	17.85 ± 0.69	39.23 ± 1.59	9.75 ± 0.39
**DOX@NMOF-PEI**	47.55 ± 2.91	7.05 ± 0.43	25.2 ± 1.28	3.73 ± 0.19
**DOX@NMOF-PEI-GA**	6.89 ± 0.38	2.067 ± 0.114	1.94 ± 0.12	0.582 ± 0.036

Table S4. Results of flowcytometry analysis of the apoptotic effect of the developed nanoformulations; DOX@NMOF and DOX@NMOF-PEI-GA as compared to the free DOX.

Sample code	Tested Dox conc. (µg/ml)	Apoptosis		Necrosis
		Early	Late	
**DOX.HCl**	5.88	16.22	7.15	2.57
**DOX@NMOF**	17.85	14.09	5.26	3.16
**DOX@NMOF-PEI-GA**	2.067	12.92	14.6	7.94
**HepG2 cells (control)**	0	0.33	0.16	1.23

Table S5. Results of DNA content analysis by the flow cytometry of the tested formulations

Sample code	Tested conc. (µg/ml)	%G0-G1	%S	%G2-M	%Pre-G1
**DOX.HCl**	5.88± 1.42	39.61	38.31	22.08	25.94
**DOX@NMOF**	71.4± 2.78	42.87	24.52	32.61	22.51
**DOX@NMOF-PEI-GA**	6.89± 0.38	37.51	44.12	18.37	35.46
**HepG2 cells (control)**	0	44.92	29.57	25.51	1.72
